# Low diversity, activity, and density of transposable elements in five avian genomes

**DOI:** 10.1007/s10142-017-0545-0

**Published:** 2017-02-11

**Authors:** Bo Gao, Saisai Wang, Yali Wang, Dan Shen, Songlei Xue, Cai Chen, Hengmi Cui, Chengyi Song

**Affiliations:** Joint International Research Laboratory of Agriculture and Agri-product Safety, College of Animal Science and Technology, Yangzhou University, 48 Wenhui East Road, Yangzhou, Jiangsu 225009 China

**Keywords:** Avian, Transposable elements, Genome size, Activity, Diversity

## Abstract

In this study, we conducted the activity, diversity, and density analysis of transposable elements (TEs) across five avian genomes (budgerigar, chicken, turkey, medium ground finch, and zebra finch) to explore the potential reason of small genome sizes of birds. We found that these avian genomes exhibited low density of TEs by about 10% of genome coverages and low diversity of TEs with the TE landscapes dominated by CR1 and ERV elements, and contrasting proliferation dynamics both between TE types and between species were observed across the five avian genomes. Phylogenetic analysis revealed that CR1 clade was more diverse in the family structure compared with R2 clade in birds; avian ERVs were classified into four clades (alpha, beta, gamma, and ERV-L) and belonged to three classes of ERV with an uneven distributed in these lineages. The activities of DNA and SINE TEs were very low in the evolution history of avian genomes; most LINEs and LTRs were ancient copies with a substantial decrease of activity in recent, with only LTRs and LINEs in chicken and zebra finch exhibiting weak activity in very recent, and very few TEs were intact; however, the recent activity may be underestimated due to the sequencing/assembly technologies in some species. Overall, this study demonstrates low diversity, activity, and density of TEs in the five avian species; highlights the differences of TEs in these lineages; and suggests that the current and recent activity of TEs in avian genomes is very limited, which may be one of the reasons of small genome sizes in birds.

**Electronic supplementary material:**

The online version of this article (doi:10.1007/s10142-017-0545-0) contains supplementary material, which is available to authorized users.

## Introduction

The elucidation of genome sequences has produced an unprecedented wealth of information about the origin, diversity, and genomic impact of repeats, and more particularly TEs, which were thought to be “junk DNA,” although long before whole genome sequencing began, it was known that these elements can sometimes account for a major proportion of genomes (Britten and Kohne 1968). We now know that, depending on the organism, the proportion of TEs in the genome can differ widely, ranging from a few percent (2.7%) of the fugu genome (Aparicio et al. 2002) to a huge proportion encompassing almost the entire genome (>80%) of maize and wheat (SanMiguel et al. 1998; Parlange et al. 2011), and they have profound effects on the structure, size, and evolution of their host genomes (Kazazian 2004). TEs make up an important part of most vertebrate genomes (Chalopin et al. 2015). However, the global contribution of TEs is variable between vertebrate lineages: for example, the genome of mammals contains many more TEs than the genome of birds (Smit 1999; Chalopin et al. 2015). Significant variability in TE content is also observed within close lineages: in teleost fish, the genome coverage of TEs is ten times higher in zebrafish than in the compact genomes of pufferfish (Chalopin et al. 2015).

The avian genome is principally characterized by its constrained size. It has been suggested that one of the reasons for the small genome is the lineage-specific erosion of repetitive elements, almost all avian genomes contained lower levels of repeat elements (~4 to 10% of each genome) than in other tetrapod vertebrates (Hillier et al. 2004; Wicker 2004; Zhang et al. 2014), such as mammal genomes, where as much as half of their genomes represent interspersed repeats derived from mobile elements (Smit 1999), and TE densities in the avian genomes are also substantially lower than that in crocodilian genomes (~27 to 37% of each genome) (Green et al. 2014), which are the closest relatives of birds.

Invaluable information concerning the density, diversity, and activity of TEs in avian genomes has recently emerged from the analyses of the draft genomes of diverse of birds, and the repeat landscapes of birds are dominated by LTR and LINE TEs (Hillier et al. 2004; Dalloul et al. 2010; Warren et al. 2010; Ganapathy et al. 2014; Zhang et al. 2014). In silico genomic mining across the avian phylogeny revealed that all nonretroviral endogenous viral elements are present at low copy numbers and in few species compared to mammals, with only endogenous hepadnaviruses widely distributed, and covering the genera alpha, beta, gamma, and epsilon retrovirus (Cui et al. 2014). However, our knowledge about these agents of genomic change across avian species, as well as the reason for low TE density in avian genomes, is still very limited. In this study, we annotated the TE landscapes of these five avian species (budgerigar, chicken, medium ground finch, turkey, and zebra finch) by using RepeatMasker (http://repeatmasker.org) and multiple de novo repeat prediction pipelines (MGEScan-non-LTR, LTRharvest, RetroTector) (Sperber et al. 2007; Ellinghaus et al. 2008; Rho and Tang 2009). By integrating analyses of these five avian species, we can perform a comprehensive analysis of TE contents across species and make inferences about the causes of low TE density in avian genomes. We investigated the abundance and distribution of TEs and highlighted the differences of TE evolution within the five avian species. Our results revealed that there was a dramatically different expansion of TE types within avian genomes, that proliferation dynamics contrasted both between TE types and between species, and we conclude that one of the reasons of low repeat density in avian genomes is due to low recent and current TE activities.

## Materials and methods

### Repeat annotation

The five avian genomes, including the genomes of chicken (Galgal4), turkey (Turkey_2.0), and zebra finch (taeGut3.2.4) were downloaded from the Ensembl Genome Browser and updated on 18 November 2015, while the gnome of budgerigar (melUnd1) and medium ground finch (geoFor1) were downloaded from the UCSC Genome Browser and updated on 13 July 2012, used for further repeat analysis. The repeat content of the avian genomes was assessed with RepeatMasker (version 4.4, http://repeatmasker.org) by using the custom library combined with RepBase database (Jurka et al. 2005) and de novo repeats identified by RepeatModeler (Version Beta 1.0.3, http://repeatmasker.org/RepeatModeler.html), MGEScan-non-LTR (Rho and Tang 2009), LTRharvest (Ellinghaus et al. 2008), and RetroTector (Sperber et al. 2007). The RepBase database of consensus repeat sequences was used to identify repeats in the genome derived from known classes of elements (Jurka et al. 2005), while RepeatModeler uses two complementary programs of RECON and RepeatScout to identify de novo repetitive sequences. The LINE retrotransposons were identified by MGEScan-non-LTR (Rho and Tang 2009), and the endogenous retroviruses were identified by LTRharvest (Ellinghaus et al. 2008) and RetroTector with default settings (Sperber et al. 2007). MGEScan-non-LTR is a computational pipeline to identify and classify the non-LTR retrotransposons in genomes; LTRharvest is an efficient software for de novo detection of LTR retrotransposons, while RetroTector is an automated recognition platform for retroviral sequences in genomes. Elements identified by LTRharvest and RetroTector programs were aligned to the domains of ENV (>480 aa), or GAG (>500 aa), or POL (>800 aa) of the reference ERVs of avian genomes to extract full ERVs. The access numbers of the reference ERVs used for alignment in GenBank are AAA19607, AAA46301, AAA46302, AAA46303, AAA46304, AAA46306, AAA46307, AAA49065, AAA62193, AAB31928, AAN38982, AAQ55054, ADO33893, AEW89630, AFA52560, AGL81187, AJG42162, BAA01499, CAA48535, CAA86524, CAC28508, CAF25154, CAF25155, EMC80838, NP_989963, Q7SQ98, XP_004950930, XP_005481887, XP_008633464, XP_009098778, XP_009928519, XP_009966720, XP_009996153, XP_010173689, XP_010219225, XP_010402058, XP_010404045, XP_010409170, XP_010720242, XP_010724325, XP_011579807, XP_011593012, and YP_004222727. The newly identified LINE and ERV elements with intact RT domains were remained for further RepeatMasker analysis, and deposited as supplementary Data 1–5. The results from the RepBase database and de novo repeats were combined and used to construct species-specific repeat libraries (supplementary Data 6–10) for the final RepeatMasker annotation. The repeat redundancies were removed based on the 80-80 rule, which considers two sequences as the same family if they could be aligned over more than 80% of their length with over 80% identity. The LINE and ERV elements (fasta-format) extracted by MGEScan-non-LTR, LTRharvest, and RetroTector are available upon request.

### Construction of phylogenetic trees

Based on an amino acid multiple alignment of the conserved RT domain from retrotransposons and reference elements, phylogenetic trees of LINE and ERV were inferred with MrBayes (Ronquist et al. 2012), applying a mixed amino acid model with a discrete gamma distribution with four rate categories and random starting trees. Two independent runs with four Markov chains each were operating for one million generations with a sampling frequency set to 100. All RT region sequences for the alignment are deposited as supplementary Data 11 and 12. Trees were drawn using Dendroscope (version 3.5.7, http://ab.inf.uni-tuebingen.de/software/dendroscope/welcome.html).

### TE age analysis

Sequence divergences of TEs from the consensus sequences, including CpG sites, which may result in older age estimates, were computed by RepeatMasker. The substitution level K was calculated with the simple Jukes-Cantor formula *K* = −300/4 × Ln(1 − *D* × 4/300) as in Abrusán et al. (2008), where *D* represents the proportion of sites that differ between the fragmented repeat and the consensus sequence. Estimates of the ages of TEs were obtained by using the equation *t* = *K*/2*r* (Kimura 1980), where *t* is the age, and *r* is the average nucleotide substitution rate for each avian species, which are 2.22 × 10^−9^, 2.00 × 10^−9^, 3.56 × 10^−9^, 2.05 × 10^−9^, and 3.44 × 10^−9^ per site per year for budgerigar, chicken, turkey, medium ground finch, and zebra finch, respectively (Zhang et al. 2014).

## Results

### Very few retrotransposons are active in the avian genomes

To identify the potential active retrotransposons in avian genomes, we applied the MGEScan-non-LTR program to extract the LINE elements. In total, 772, 262, 42, 46, and 30 “ORF-preserving” LINEs were identified in the genomes of the budgerigar, chicken, medium ground finch, turkey, and zebra finch, respectively, and these elements were initially classified into three clades (CR1, R2, and RTE) by the MGEScan-non-LTR pipeline. The majority of them are CR1 elements, only nine R2 elements in zebra finch, and two R2 elements in medium ground finch, and one RTE element in budgerigar were detected. Most of the LINE retrotransposons are defective; only 55 CR1s in budgerigar, 14 CR1s in chicken, 1 R2 in medium ground finch, and 6 R2s in zebra finch contain intact RT domains (Table 1). The CR1 elements with both ORF1 and long ORF2 (>600 aa) were retained and designated as full LINE, which may be active. Only one full CR1 was detected in the lineages of budgerigar and chicken, no full CR1 in the other three avian genomes was found. Five and one full R2s with intact ORF2 in budgerigar and zebra finch were detected, respectively (Table 1), suggesting that these R2 elements may be active as well.

**Table 1:** Initial classification of LINEs in the avian genomes by MGEScan-non-LTR

Clade		Budgerigar	Chicken	Medium ground finch	Turkey	Zebra finch
Total		772	262	42	46	30
CR1	Total	771	262	40	46	21
	Elements with intact RT	55	14	0	0	0
	Elements with ORF1	6	2	0	0	0
	Elements with ORF2 (>600 aa)	11	8	0	0	0
	Full elements	1	1	0	0	0
R2	Total	0	0	2	0	9
	Elements with intact RT	0	0	1	0	6
	Elements with ORF1	0	0	–	0	–
	Elements with ORF2 (>600 aa)	0	0	1	0	5
	Full elements	0	0	1	0	5
RTE	Total	1	0	0	0	0
	Elements with intact RT	0	0	0	0	0

**Fig. 1:**
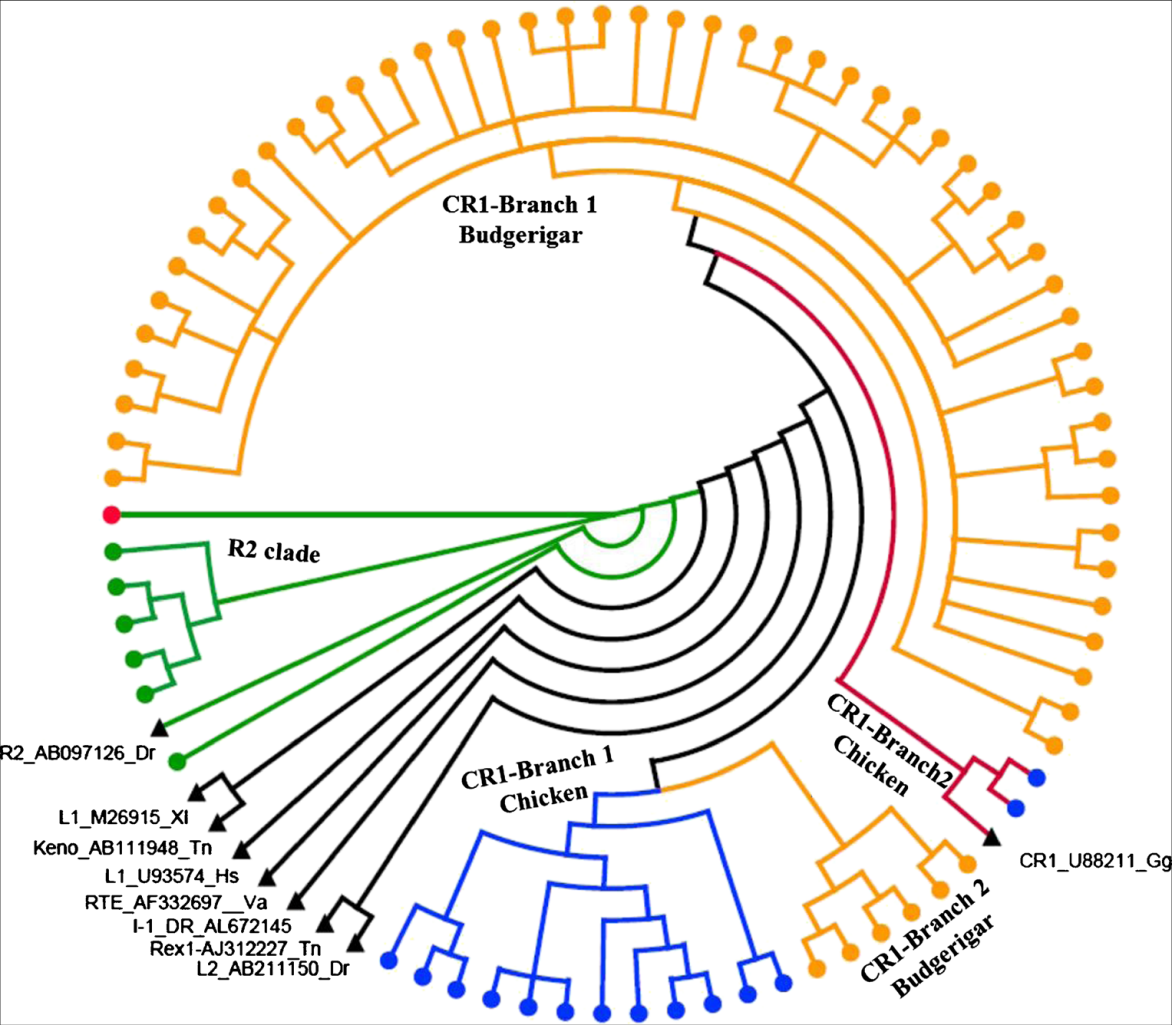
Phylogenetic position of CR1 and R2 clades in the avian genomes relative to previously described families. The nodes of sequences from budgerigar, chicken, medium ground finch, and zebra finch are shown as *yellow*, *blue*, *red*, and *green dots*, respectively, and the nodes of reference elements are indicated by *black triangles* (color figure online)

Phylogenetic analysis confirmed that these LINE elements with intact RT domain belong to CR1 and R2 clades of LINEs in the avian species, and the CR1 clade is very diverse in the family structure and both of chicken and zebra finch CR1 elements were further classified into two branches, while the R2 clades represent relatively little family structure compared with the CR1 clade, and only a few families in the medium ground finch genome and one family in the zebra finch lineage were detected (Fig. 1).

ERVs in the five avian genomes were extracted using LTRharvest and RetroTector pipelines. With the LTRharvest program, we detected 523, 788, 1301, 523, and 6220 LTR elements within the budgerigar, chicken, medium ground finch, turkey, and zebra finch genomes, respectively; using the RetroTector with the default baseline quality threshold of 250, we identified 960, 887, 1068, 293, and 3388 ERV-derived elements within the budgerigar, chicken, medium ground finch, turkey, and zebra finch genomes, respectively (Table 2). Elements containing the conserved ENV (>480 aa), or GAG (>500 aa), or POL (>800 aa) domain of ERV were retained, and the ERVs containing three domains were designated as full ERV. We found that the zebra finch genome has more elements containing ERV domains, followed by the chicken genome, with very few elements containing ERV (ENV, or GAG, or POL) domains detected in the other three avian genomes. In total, only one full ERV was detected in chicken with both pipelines and may be active, but no full ERV was detectable in the other four avian genomes (Table 2).

**Table 2:** Characteristics of ERVs in the avian genomes

Species	LTRHarvest					RetroTector				
	Elements identified	Elements with ERV domain			Full ERVs	Elements identified	Elements with ERV domain			Full ERVs
		POL	GAG	ENV			POL	GAG	ENV	
Budgerigar	523	1	1	0	0	960	2	2	1	0
Chicken	788	22	31	3	1	887	30	41	6	1
Medium ground finch	1301	3	0	0	0	1068	5	2	0	0
Turkey	523	1	1	0	0	293	1	1	1	0
Zebra finch	6220	13	12	9	0	3388	53	24	38	0

The LTRs identified by LTRHarvest and RetroTector programs were aligned with the ENV, GAG, and POL amino acid sequences of reference ERVs of avian genomes. The elements with the conserved ENV (>480 aa), or GAG (>500 aa), or POL (>800 aa) domain of ERV were retained. The ERVs containing all three domains were designated as full ERVs

**Fig. 2:**
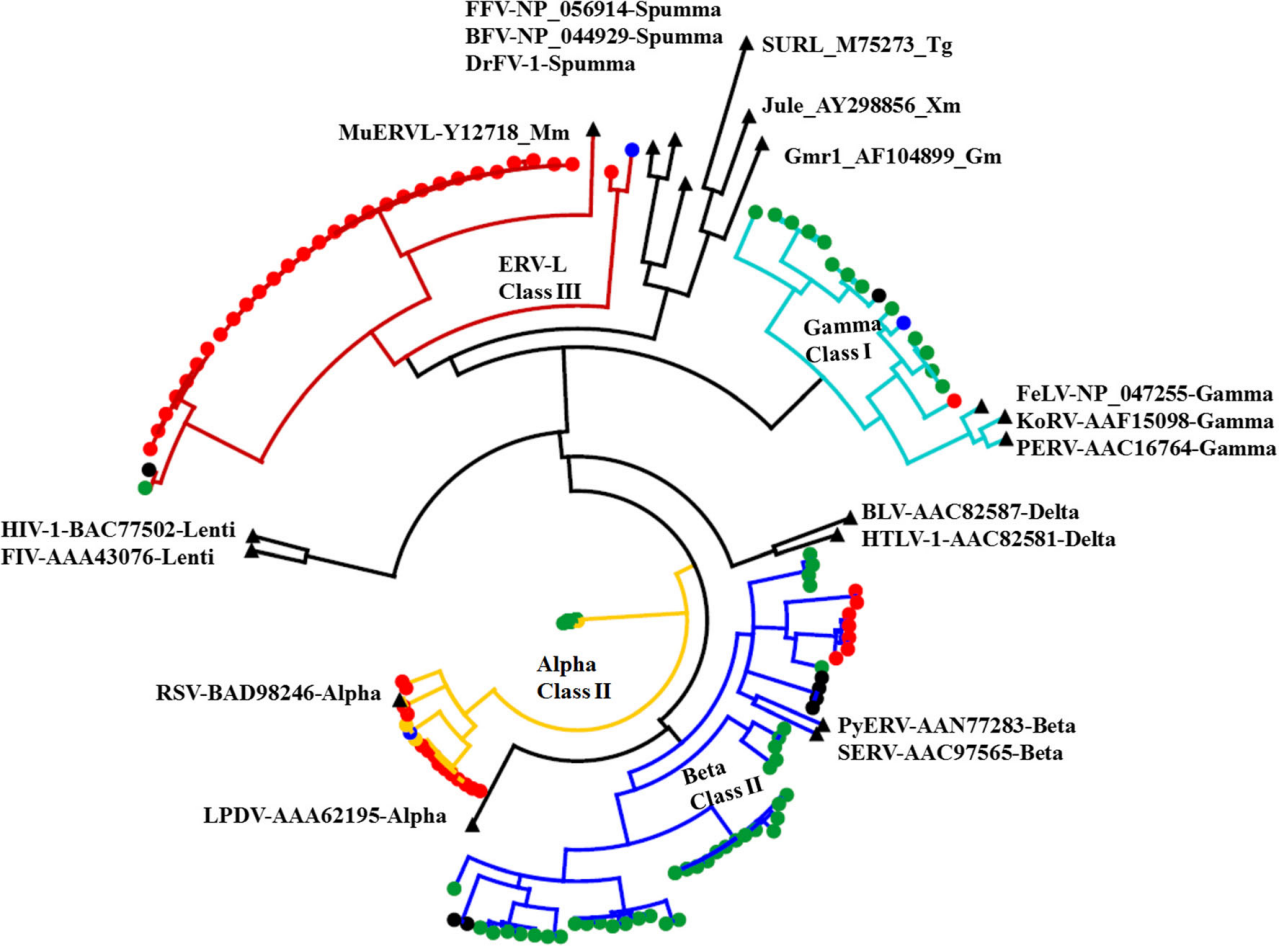
The RT phylogenetic tree of ERVs in the avian genomes. The nodes of sequences from budgerigar, chicken, medium ground finch, turkey, and zebra finch are shown as *blue*, *red*, *black*, *yellow*, and *green dots*, respectively, and the nodes of reference elements are indicated by *black triangles*. Abbreviation lists of reference endogenous retrovirus: BLV, bovine leukemia virus; HTLV-1, human T-lymphotropic virus 1; FIV, feline immunodeficiency virus; HIV-1, human immunodeficiency virus 1; KoRV, koala retrovirus; PERV, pig endogenous retrovirus; PyERV, python endogenous retrovirus; FeLV, feline leukemia virus; BFV, bovine foamy virus; FFV, feline foamy virus; DrFV-1, *Danio rerio* foamy virus type 1; MuERV-L, murine endogenous retrovirus-leucine; SERV, simian endogenous retrovirus. The Jule, SURL, and Gmr1 are reference elements of GYPSY/LTR retrotransposons (color figure online)

These ERVs with POL domain were classified into four clades (alpha, beta, gamma, and ERV-L) and belong to three classes of ERV (ERV1, ERV2, and ERV3) by phylogenetic analysis. However, these ERVs are uneven distributed in birds, most of them distribute within the chicken and zebra finch lineages, diverse gamma ERV families (ERV1) present in the lineage of zebra finch, with only one gamma ERV family in each genome of budgerigar, chicken, and medium ground finch, while many ERV-L (ERV3) families distribute in the lineage of chicken, with only one ERV-L family in each genome of budgerigar, medium ground finch, and zebra finch. Abundant alpha and beta ERV families (ERV2) distribute in the chicken and zebra finch lineages with only six beta ERV families in the medium ground finch lineage and two alpha ERV families in the turkey genome (Fig. 2).

### LINEs and LTRs dominate the repeat landscapes of the avian genomes

The total interspersed repeats of the five avian genomes were identified and classified by combining analyses with the RepBase library and de novo RepeatModeller program as described in the “Materials and methods” section. A summary of the main groups of the total interspersed repeats is listed in Table 3. Generally, the TE contents in the five avian genomes are similar and occupy 9.50, 10.55, 7.67, 8.58, and 9.21% of the budgerigar, chicken, medium ground finch, turkey, and zebra finch genomes, respectively (Table 3), which are substantially lower than that of mammals (Smit 1999; Chalopin et al. 2015). Comparison of the abundance distributions of TEs across the five avian genomes revealed contrasting proliferation profiles both between TE types and between species. The avian genomes were dominated by LINE and LTR repeats, while DNA and SINE repeats are quite rare and display very low abundance (Table 3). LINEs represent the most abundant elements in most investigated birds except zebra finch, comprising 7.38, 7.05, 3.69, and 6.31% of the budgerigar, chicken, medium ground finch, and turkey genomes, respectively. LTRs are the second major repeat types and comprise 1.43, 1.92, 3.00, and 1.05% of the budgerigar, chicken, medium ground finch, and turkey genomes, respectively. In zebra finch, LTRs represent the most abundant elements at 4.28% of genome coverage, with LINEs the second major repeat type, at 3.68% of genome coverage. Compared with the LTRs and LINEs, the DNA repeats occupy the smaller portion of the bird genomes and represent only 0.28, 1.02, 0.27, 0.98, and 0.19% of the budgerigar, chicken, medium ground finch, turkey, and zebra finch genomes, respectively. The SINE elements exhibit extremely low density and comprise only 0.06–0.08% of these avian genomic sequences (Table 3).

**Table 3:** Genome coverage of TEs in the avian genomes

Types of repeat	Genome coverage (%/bp)				
	Budgerigar	Chicken	Medium ground finch	Turkey	Zebra finch
SINEs	0.08/836,533	0.06/667,119	0.06/598,523	0.07/611,145	0.07/854,724
LINEs	7.38/80227642	7.05/72,829,959	3.69/38,406,325	6.31/59,107,954	3.68/44,965,242
CR1	7.33/79,702,384	7.00/72,332,909	3.65/38,057,724	6.28/58,757,159	3.64/44,473,606
Other LINEs	0.05/525,258	0.05/497,050	0.03/348,601	0.04/350,795	0.04/491,636
LTRs	1.43/15,568,639	1.92/19,839,041	3.00/31,259,772	1.05/9859507	4.28/52,374,285
ERV total	1.43/15,072,290	1.91/19,772,771	3.00/31,233,048	1.05/9,835,758	4.20/51,315,552
ERV1	0.17/1,883,003	0.32/3,349,074	0.43/4,482,424	0.06/527,298	0.76/9,261,943
ERV2	0.01/87,475	0.19/1,921,798	1.19/12,402,067	0.01/53,503	2.01/24,599,045
ERV3	1.21/13,202,841	1.40/14,501,899	1.37/14,231,337	0.99/9,254,957	1.35/16,550,924
Other LTRs	0.00/42,075	0.01/66,270	0.00/26,724	0.00/23,749	0.09/1,058,733
DNA	0.28/3,082,949	1.02/10,499,572	0.27/2,853,091	0.98/9,132,769	0.19/2,359,685
Unclassified	0.33/3,544,343	0.50/5,153,202	0.65/6,761,019	0.17/1,565,220	0.99/12,067,610
Total interspersed repeats	9.50/103,260,106	10.55/108,988,894	7.67/79,878,730	8.58/80,276,595	9.21/112,621,546

### Low diversity of LINE and LTR TEs in the avian genomes

**Fig. 3:**
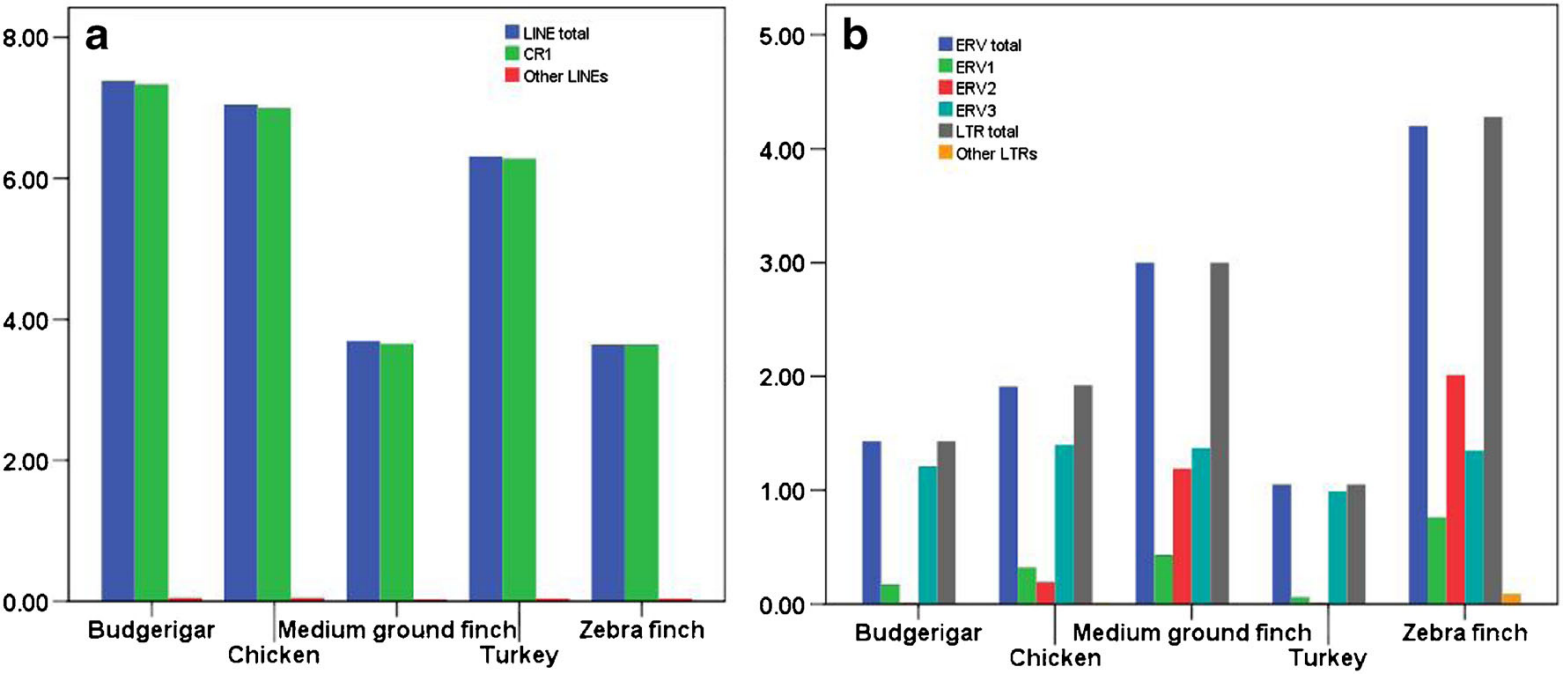
Genome coverages of LINE and LTR TEs in the five species of birds

Although LINE and LTR repeats are the major TEs in these genomes, closer analysis revealed that the diversity of LINE and LTR TEs at clade (superfamily) level is very low and a striking differential accumulation of TE clades within both LINE and LTR repeat types was observed (Table 3 and Fig. 3). The predominant clade of LINEs is CR1 in all five avian species investigated, which comprises 7.33, 7.00, 3.65, 6.28, and 3.64% of the budgerigar, chicken, medium ground finch, turkey, and zebra finch genomes, respectively, while the other clades represent an extremely low proportion of these genomes, and in total occupy less than 0.05% of genomes (Table 3 and Fig. 3a). The major clade of LTRs is ERV in all five avian species investigated, which comprises 1.43, 1.91, 3.00, 1.05, and 4.20% of the budgerigar, chicken, medium ground finch, turkey, and zebra finch genomes, respectively, while the other clades of LTRs exhibit extremely low density, and in total represent less than 0.1% of the avian genomes (Table 3 and Fig. 3b). Dramatically differential expansions of ERV classes across the avian genomes were also observed. ERV3 is the most abundant class of ERVs within most genomes investigated here and comprises from 0.99 to 1.40% of their sequences, while ERV1 exhibits low proliferation during the evolution histories and represents less than 0.8% of most of these genomes. ERV2 has experienced a substantial expansion only within the medium ground finch and zebra finch genomes and occupies 1.19 and 2.01% of genomic sequences, respectively (Table 3 and Fig. 3b).

### Low recent and current activity of TEs in the avian genomes

**Fig. 4:**
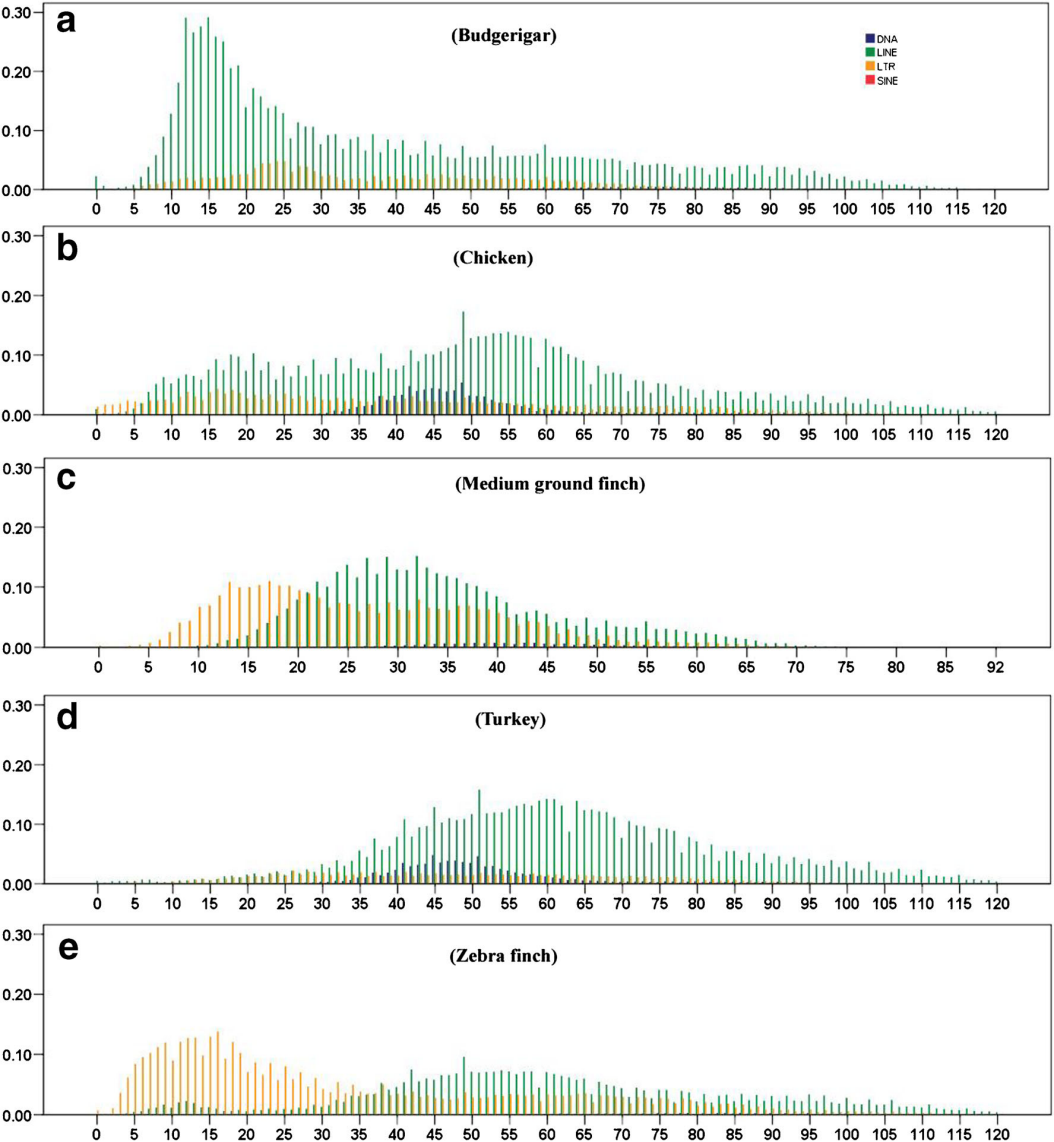
Divergence distribution of TE types (LINE, LTR, SINE, and DNA TEs) in the budgerigar (**a**), chicken (**b**), medium ground finch (**c**), turkey (**d**), and zebra finch (**e**) genomes. The *x*-axis represents the insertion time (million years), and the *y*-axis represents the percentage of the genome comprised of repeat classes (%)

**Fig. 5:**
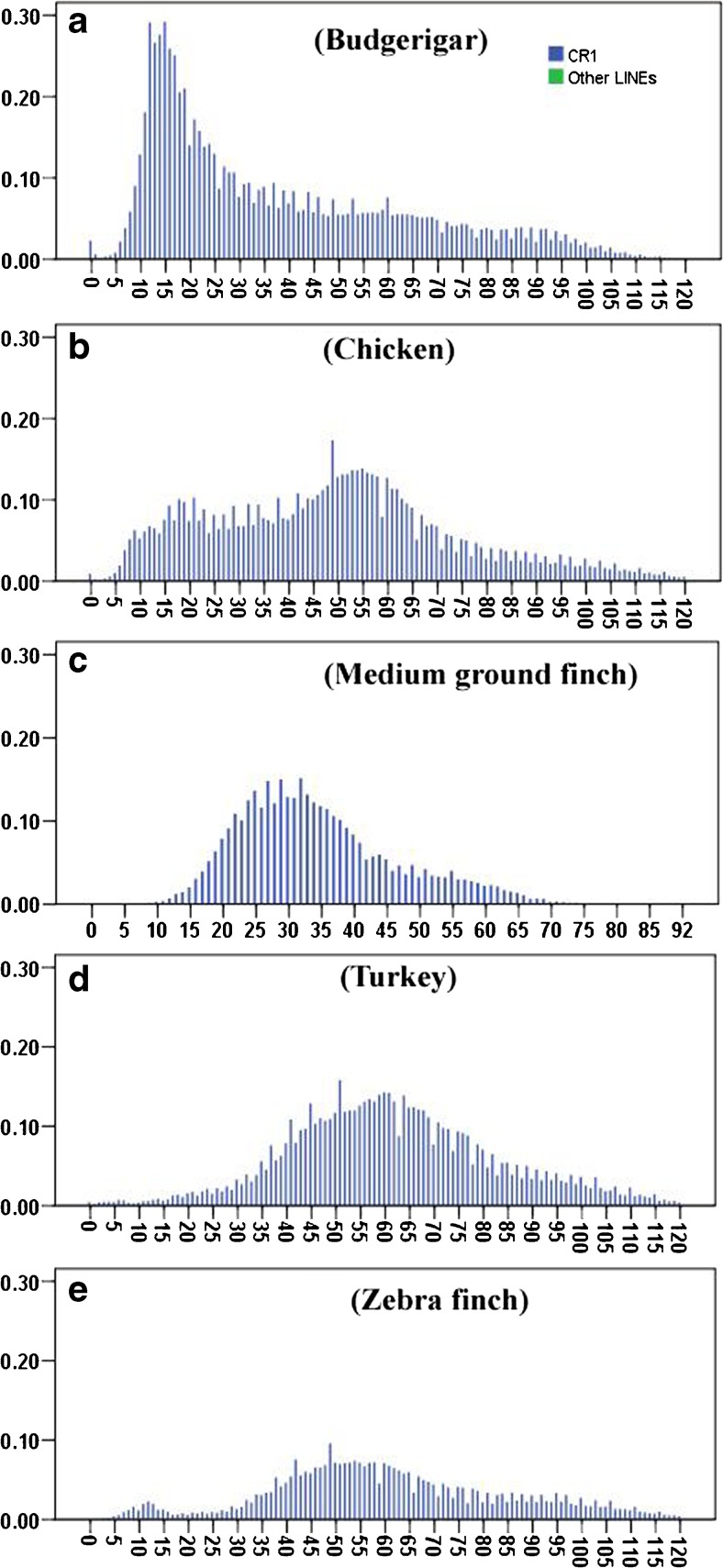
Divergence distribution of LINE clades (CR1 and other LINE clades) in the budgerigar (**a**), chicken (**b**), medium ground finch (**c**), turkey (**d**), and zebra finch (**e**) genomes. The *x*-axis represents the insertion time (million years), and the *y*-axis represents the percentage of the genome comprised of repeat classes (%)

**Fig. 6:**
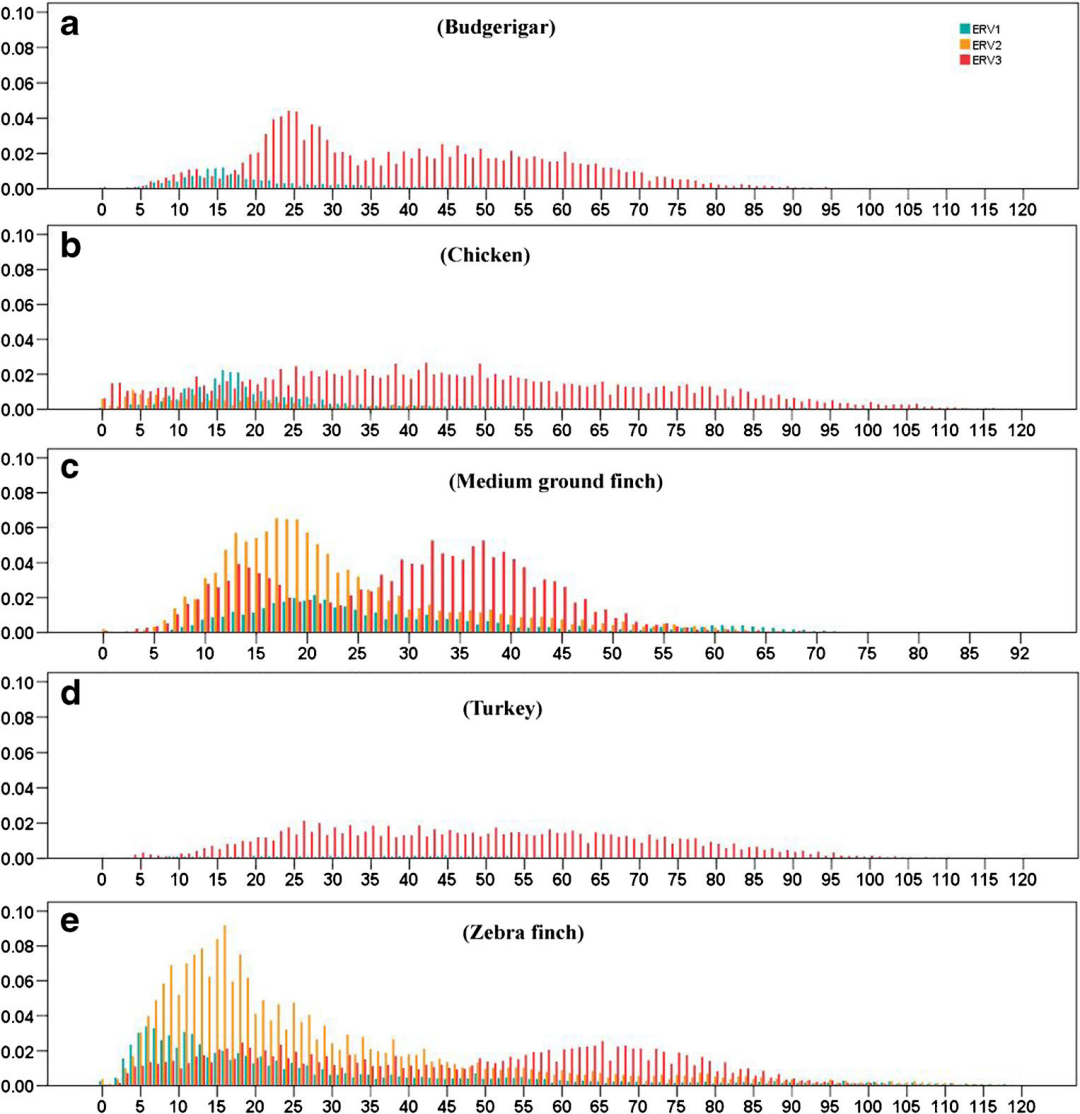
Divergence distribution of ERV Classes (ERV1, ERV2, and ERV3) in the budgerigar (**a**), chicken (**b**), medium ground finch (**c**), turkey (**d**), and zebra finch (**e**) genomes. The *x*-axis represents the insertion time (million years), and the *y*-axis represents the percentage of the genome comprised of repeat classes (%)

The divergence of TE sequences was used to calculate the age of insertion of each class and subclass of TEs, and a graph of their distribution in time was built. Generally, the age distributions of TEs revealed a low recent and current activity of TEs across most of these avian genomes (Fig. 4). Overall, the LINE and LTR retrotransposons in these genomes have been active over a relatively longer time period, and exhibit relatively stronger activity during the evolution of genomes, when compared with DNA and SINE repeat types. However, most LINE TEs here, except budgerigar, are ancient copies and show major insertions of relatively old age with a substantial decrease in activity in the last 20 million years (My) (Fig. 4), while LINEs in budgerigar exhibit a recently sharp expansion between 5 and 25 My, followed by a significant decrease of activity (Fig. 4a). Weakly recent (5–10 My) activities of LINEs in chicken and zebra finch were observed (Fig. 4b. e), but the current activity of LINEs is very limited in all avian species investigated, as shown by a very low proportion of LINE copies that are less than 5 My (Fig. 4). Most LTRs are ancient copies and show weak proliferation during the evolution of budgerigar, chicken, and turkey genomes, while LTRs in medium ground finch and zebra finch exhibit a recent expansion between 5 and 25 My with a peak of activity around 13 My (Fig. 4c, e), which is different from the other three avian species. Current activity of LTRs may be maintained in chicken and zebra finch, but almost distinct in the other three avian lineages, as shown a small proportion of LTR copies in chicken and zebra finch, and extremely low copies of LTRs in the other three lineages in the last 5 My (Fig. 4). While the activities of DNA and SINE TEs within all these avian genomes are very limited during their whole evolution histories, and the activities have been extinct at least 30 My within the avian lineage (Fig. 4), only one round of expansion of DNA repeats between 35 and 65 My within chicken and turkey lineages was observed (Fig. 4b, d), the accumulation of this TE class is extremely low in the other three lineages (Fig. 4a, c, e). In-depth analysis of the age distribution between the CR1 clade and the other LINE clades revealed that CR1 dominates the evolution of LINEs in these avian, and weakly recent activities of CR1 in chicken and zebra finch, and sharply recent burst of CR1 in budgerigar were observed. The activities of all the other LINE clades were extremely low and hard to detect (Fig. 5). Contrasting proliferation dynamics of ERV classes of LTRs were also observed in the avian genomes (Fig. 6). ERV3 has experienced a relatively older, longer, and medium expansion in all the five avian genomes, and followed by a substantial decrease in activity in the last 5 My, except chicken and zebra finch (Fig. 6), where young activity was observed, as shown a small proportion of ERV3 copies in the last 5 My (Fig. 6a, e), while ERV2 exhibits recently expansion only within medium ground finch and zebra finch, with peaks of activity at 16 Ma, and the activity of ERV2 in the budgerigar, chicken, and turkey genomes is very weak (Fig. 6). ERV1 has experienced one round weak expansion around 15, 15, and 20 My in budgerigar, chicken, and medium ground finch, respectively (Fig. 6a–c), while ERV1 in zebra finch exhibits a young burst in the last 15 My with a peak at 6 My (Fig. 6e), and its activity is extremely weak in the turkey lineage (Fig. 6d).

## Discussion

In this study, we investigated the abundance, diversity, and activity distribution of TEs among five avian species. Compared with the other vertebrates, the avian genomes represent a clearly different accumulation profile of TEs and show a significant difference in the classes of TEs present, their fractional representation in the genome, and the level of TE activity. The estimated fraction of repeats (about 10%) within the avian genomes in this study is substantially lower than that in the most investigated vertebrates together with fish including zebrafish (about 55%) (Howe et al. 2013) and carp (31.3%) (Xu et al. 2014), reptiles including lizard (34.4%) (Alföldi et al. 2011) and frog (34.5%,) (Hellsten et al. 2010), and mammalian genomes (about 45%) (Chalopin et al. 2015; Pefanis et al. 2015). The coverage of repeat contents in the chicken (10.45%) and zebra finch (9.01%) in this study are higher than the early TE annotations of chicken (8.5%) (Hillier et al. 2004) and zebra finch (7.7%) (Warren et al. 2010). The disagreement may be due to the underestimate because the genome is far from complete and repeat dense regions are underrepresented in the previous draft assembly.

The evolutionary dynamics of TEs in vertebrates are drastically different. The genomes of mammals contain a limited number of types in great abundance, while the genomes of reptile and fish represent relatively higher diversity and activity of TEs (Chalopin et al. 2015). Our study distinctly shows that the levels of TE diversity, activity, and density in birds are much lower than those seen for reptile, most fish, and mammalian genomes. Although the densities of LINE and LTR TEs in fish and reptile genomes vary significantly, the diversities of these TE types are extremely high and most LINE (CR1, L1, L2, R2, RTE, I, REX) and LTR (BEL/PAO, Copia, DIRS, ERV, Gypsy, Ngaro) clades were detected within reptile and fish genomes, and the activities of these TEs are high as well as indicated with rich intact families detected in each clade (Alföldi et al. 2011; Howe et al. 2013; Chalopin et al. 2015). The high diversity of DNA transposons was already noted in reptiles and fish (Hellsten et al. 2010; Alföldi et al. 2011; Howe et al. 2013; Chalopin et al. 2015). In contrast, we found that the diversity of retrotransposons in avian lineages is low, and the avian genomes were dominated by CR1 clade of LINEs and ERVs of LTRs, and all other clades of LINEs and LTRs did not show substantial accumulation, representing a very small portion of each genome. This also contrasts with most mammals, where the expansion of the genome is dominated by L1 retrotransposons (Smit 1999). Although the diversity of LINEs at the clade (superfamily) level in avian species is low with CR1 dominating the evolution of avian genomes, we found that the diversity of CR1 at family level is high, at least two distinct branches with many families were identified in chicken and zebra finch. This conclusion is in good agreement with the previous study, which revealed that CR1s in birds evolve into many subtypes at different periods of bird evolution, and for each CR1 subtype, there was one limited period of activity (Kriegs et al. 2007). In addition, several new subfamilies different from other previously described avian CR1 subfamilies were also identified in waterfowl, and despite the possible lack of an active CR1 in chicken, at least one of these subfamilies in this order was suggested to be likely active (St. John et al. 2005; John and Quinn 2008). On the other hand, these insertion polymorphisms of these CR1 retrotransposons also used as phylogenetic markers to elucidate the evolution of bird and reflecting the rapid diversification of these birds (Kaiser et al. 2007; Treplin and Tiedemann 2007; Liu et al. 2012; Suh et al. 2012). While DNA TEs just occupy about 1% or less of the avian genomes, and SINEs comprise 0.06–0.08%, the activity, diversity, and density of DNA and SINE TEs are also substantially lower than that in most vertebrates (Chalopin et al. 2015).

The age distribution analysis revealed that all DNA and SINE TEs are fossils and the activity has been extinct for at least for 30 My; only CR1 of LINEs and ERVs of LTRs show limited recent activity in the avian genomes, and some elements may still be currently active. Previous study reveals that three major peaks of CR1 activity were observed in the evolution of gamebirds, including megapodes, currassows, guinea fowl, New and Old World quails, chicken, pheasants, grouse, and turkeys, based on the analysis of 22 known CR1 subtypes, and H2, F0, B2, F2, D2, and C2 subtypes of CR1 represent the youngest peaks (Kriegs et al. 2007); the evolution dynamics of these subtypes were investigated within neoavian birds as well (Matzke et al. 2012). Here, our data revealed the current activity of these clades is very restricted due to very few full elements with intact ORFs remaining in the avian genomes as well as very low levels of recent activity reflected by the divergence distribution analysis (Fig. 5), although the intact LINE and ERV elements may be underestimated due to short read sequencing and low coverage in the current assemblies. Across all five investigated avian genomes, we only found several intact LINE elements (one CR1 in chicken, five R2 in budgerigar, and one R2 in zebra finch), which is in agreement with previous studies in chicken (Hillier et al. 2004; Wicker 2004; Wicker et al. 2005), and the full-length R2 elements in zebra finch genome (Kordis 2010) and RTE elements in diverse avian species including budgerigar (Suh et al. 2016) already noted previously; furthermore, very recently study revealed that R2 is distributed among almost all of the major groups of birds, except Galloanseres (chickens and ducks) (Kojima et al. 2016). The low or lost activity of LINEs (CR1) also explains the extreme low abundance of SINEs in avian genomes since SINE expansion depends on the partners (LINEs) (Kajikawa and Okada 2002). Although the endogenous retroviruses distributed widely across avian species, the recent activity of ERVs is mainly restricted in chicken and zebra finch lineages, and the other three investigated avian species show a significant decrease of ERV activity in the last 5 My. Further analysis revealed that only one full ERV was identified in chicken, and no full ERVs in the other four avian genomes were detectable. These data indicated that most ERVs in avian genomes are degenerate and inert. In total, the current activity of LINE and LTR TEs in avian genomes is very low due to very few intact elements.

In most vertebrates with high TE contents, including mammals, frog, lizard, and some fishes (such as zebrafish and medaka), recent and current activities of TEs, which are indicated by high copies of intact and active elements within genome, play important role in the expansion of genome size and are the major contributors to the high density of TEs in genomes (Hellsten et al. 2010; Alföldi et al. 2011; Howe et al. 2013; Chalopin et al. 2015; Gao et al. 2016). Many intact and putatively active retrotransposons (LINE and LTR families) and DNA transposons (Tc1, hAT, etc.) were identified in the frog, lizard, and fish genomes (Hellsten et al. 2010; Alföldi et al. 2011; Howe et al. 2013; Chalopin et al. 2015; Gao et al. 2016), while, in the mammal, over 100 intact L1s in the human genome and over 3000 intact L1s in the mouse genome as mammals were identified (Goodier et al. 2001; Brouha et al. 2003). On the contrast, the current study revealed very few intact TEs present in the avian genomes, and most of TEs are ancient copies, which indicated that the recent and current activities are very limited. Thus, the low recent and current activities of TEs are inferenced as one of the reasons for the small genome of bird.

## Electronic supplementary material

Data 1 The new repeats in budgerigar identified by multi-pipelines (FAS 386 kb)

Data 2 The new repeats in chicken identified by multi-pipelines (FAS 438 kb)

Data 3 The new repeats in medium ground finch identified by multi-pipelines (FAS 61 kb)

Data 4 The new repeats in turkey identified by multi-pipelines (FAS 14 kb)

Data 5 The new repeats in zebra finch identified by multi-pipelines (FAS 580 kb)

Data 6 The repeat library in budgerigar for RepeatMasker annotation (FAS 647 kb)

Data 7 The repeat library in chicken for RepeatMasker annotation (FAS 709 kb)

Data 8 The repeat library in medium ground finch for RepeatMasker annotation (FAS 384 kb)

Data 9 The repeat library in turkey for RepeatMasker annotation (FAS 252 kb)

Data 10 The repeat library in zebra finch for RepeatMasker annotation (FAS 1113 kb)

Data 11 The fasta-formatted alignments of LINE RT domains (FAS 40 kb)

Data 12 The fasta-formatted alignments of ERV RT domains (FAS 28 kb)
